# Extraction of Corrosion Damage Features of Serviced Cable Based on Three-Dimensional Point Cloud Technology

**DOI:** 10.3390/ma18153611

**Published:** 2025-07-31

**Authors:** Tong Zhu, Shoushan Cheng, Haifang He, Kun Feng, Jinran Zhu

**Affiliations:** 1 Research Institute of Highway, Ministry of Transport, Beijing 100088, China; ztong2024@163.com; 2 Faculty of EAE, Anglia Ruskin University, Peterborough PE1 5BW, UK; 3 College of Civil Engineering and Architecture, Hebei University, Baoding 071002, China

**Keywords:** stay cable, high-strength steel wire, pitting corrosion, 3D laser scanning

## Abstract

The corrosion of high-strength steel wires is a key factor impacting the durability and reliability of cable-stayed bridges. In this study, the corrosion pit features on a high-strength steel wire, which had been in service for 27 years, were extracted and modeled using three-dimensional point cloud data obtained through 3D surface scanning. The Otsu method was applied for image binarization, and each corrosion pit was geometrically represented as an ellipse. Key pit parameters—including length, width, depth, aspect ratio, and a defect parameter—were statistically analyzed. Results of the Kolmogorov–Smirnov (K–S) test at a 95% confidence level indicated that the directional angle component (θ) did not conform to any known probability distribution. In contrast, the pit width (b) and defect parameter (Φ) followed a generalized extreme value distribution, the aspect ratio (b/a) matched a Beta distribution, and both the pit length (a) and depth (d) were best described by a Gaussian mixture model. The obtained results provide valuable reference for assessing the stress state, in-service performance, and predicted remaining service life of operational stay cables.

## 1. Introduction

Cable-stayed bridges are the main structural forms of modern long-span bridges due to their good spanning performance, beautiful shape, and convenient construction methods [1,2,3,4,5]. As one of the key components of cable-stayed bridges, cables are often susceptible to corrosive environments. Bridge inspections have shown that the high-strength steel wires in the cables are often corroded and that many of the cables of the cable-stayed bridges have been replaced [6,7,8]. For the microenvironment inside the cable, the temperature difference between day and night will cause water vapor inside the cable to condense into water droplets and, accompanied by the erosion of a corrosive medium, form a corrosive microenvironment. Studies have shown that corrosion pits are often formed in harsh corrosion microenvironments inside bridge cables [9]. In addition, shields and anchors that are often damaged or poorly sealed allow access to cables in corrosive environments [10].

The corrosion of high-strength steel wire is mainly manifested in two forms: uniform corrosion and pitting corrosion. Uniform corrosion leads to a decrease in the cross-sectional area of the wire, which in turn leads to a decrease in its ultimate load-bearing capacity [11]. Corrosion pitting can cause local stress concentrations [12,13] that can accelerate fatigue crack propagation over time, especially under long-term loading conditions [14,15,16]. This greatly shortens the remaining life of the cable and poses a serious risk to the operational safety of the bridge. Therefore, the corrosion characteristics of cable-stayed high-strength steel wires should be carefully considered when conducting comprehensive safety assessments of in-service cable-stayed bridges, as well as when performing mechanical property analysis and remaining service life prediction [17,18,19,20]. Since the corrosion of the steel wire has a great influence on the mechanical properties of the steel wire, statistical modeling of the corrosion is an important issue. Wire corrosion pits vary greatly in shape. They are usually shaped like a disc, cone, or hemisphere [21]. Therefore, the quantitative study of the morphological characteristics of the corrosion pits of high-strength steel wires is an important foundation for revealing the deterioration of high-strength steel wires.

A great deal of research has been performed on the uniform corrosion and pitting of metals, the mechanisms of corrosion, and the statistical characteristics of the corrosion depth of the corroded surface. The pitting corrosion of stainless steel in chloride solution and its mechanism have been studied in depth, including nucleation and propagation [22,23,24,25]. However, relatively few studies have been conducted on the corrosion pit model in steel wire. Pitting is a type of local damage to bridge structures. Digital image processing has been used to assess pitting corrosion [26,27] and detect pitting areas in concrete, steel, or other metal samples [28,29,30]. Researchers used image acquisition techniques to collect images of corroded surfaces to analyze the crater morphology on a 2D plane [31]. Vincent [32] studied grayscale reconstruction for image filtering and segmentation tasks. Choi [33] proposed a symbiotic matrix approach for the automatic identification of corroded regions, categorizing the process into three distinct feature domains: color, texture, and shape. Thanks to the continuous advancement of computer imaging technology, advanced non-contact 3D laser scanning technology has been widely used to simulate the morphological and statistical properties of corrosion pits, providing a quantitative framework for describing their geometric characteristics and mechanical effects [34]. This technology has allowed for the establishment of a more accurate corrosion model for the steel wires of high-strength cables. This technique was able to measure the depth of the corrosion pit [31]. Fang et al. [35] obtained the pitting depth of the high-strength steel wire surface by meshing the point cloud data of the 3D scan of the high-strength steel wire surface, traversing the mesh to identify the connected corrosion meshes and treating them as separate corrosion pits. Xu et al. [36,37] obtained the three-dimensional surface morphology of corroded steel plate by using 3D scanning technology and a reverse reconstruction method, proposing an evaluation index of corrosion degree based on surface morphology. Yuan et al. [38] detected and represented the actual corrosion and its distribution on several steel wire surfaces of the replaced boom. Li et al. [39] performed a statistical analysis of the corrosion characteristics of old cable and found that the uniform corrosion depth obeys a lognormal distribution and gradually decreases as the wire moves from the outside to the inside of the cable body. Chen et al. [40] analyzed the statistical characteristics of the corrosion morphology of steel wires under different strain levels and corrosion ages through accelerated corrosion tests, establishing the probability density functions of pit depth, corrosion radius, and corrosion center distance.

In the technical fields of image processing and structural engineering damage detection, accurate feature extraction and reliable error analysis are key to improving the performance of the method. Binarization processing, as a key step in image processing, can effectively simplify image information, highlight target characteristics, and lay the foundation for subsequent detection and classification tasks. Among many binary algorithms, the Otsu method has shown good performance in object detection in complex environments such as low light due to its adaptive threshold determination. Harvey et al. [41] proposed a vision-based accelerometry method that combines the thresholding method with LED targets to measure structural acceleration. He et al. [42] proposed a detection method to achieve accurate image classification by tracking the thresholded LED spots in indoor low-illumination environments using the Otsu method. The images first undergo Poisson noise processing and bilateral filtering, and then image segmentation is performed using the Otsu algorithm. Statistical testing methods provide a rigorous quantitative basis for data analysis, facilitating the differentiation of error types and enhancing the credibility of results in complex detection processes. As a classic non-parametric statistical method, the Kolmogorov–Smirnov (K-S) test offers strong support for the systematic classification and analysis of various types of errors. Ruffels et al. [43] employed the Kolmogorov–Smirnov test as a statistical tool to effectively distinguish and categorize various types of errors that may occur during the model-free damage detection process of laboratory bridges. This has contributed to more precise and efficient damage assessment methodologies.

A large number of studies have obtained the statistical characteristics of the corrosion morphology of steel wires through accelerated corrosion tests, but there is a slight gap between accelerated corrosion and the actual operating state. Few studies have been conducted on the corrosion of service cable wires. In addition, the corrosion damage and mechanical properties of steel wires in actual engineering scenarios are not thoroughly analyzed.

In order to solve this shortcoming, this paper statistically characterizes the parameters of corrosion pits on service-exposed steel wires. This study’s main contributions are as follows:

(1) The corrosion wire specimens analyzed in this study were sourced from cable replacements in a 27-year-service-life cable-stayed bridge, rather than artificially accelerated corrosion samples. This provenance ensures the resultant data exhibits superior field-relevance and authentic degradation characteristics reflective of actual infrastructure service conditions.
(2) Advanced non-contact 3D laser scanning technology was utilized to reconstruct morphological models of corroded steel wires, thereby characterizing the surface features of corroded wires. The implemented methodology has undergone validation and exhibits high measurement accuracy.
(3) Kolmogorov–Smirnov (K–S) tests were employed to analyze the probability distribution models of corrosion characteristics.

## 2. Engineering Background and Methodology

### 2.1. Engineering Background

**Figure 1:**
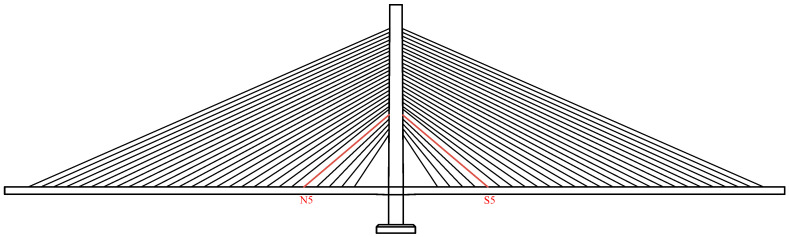
Schematic diagram of the change.

In this study, the sample originated from a cable-stayed bridge in Anhui Province, China. The bridge is a single-tower single-cable surface prestressed concrete cable-stayed bridge that was completed in 1996. The bridge has been in operation for 27 years, during which time no cable has been replaced. In 2022, the S5 and N5 cables of the bridge were replaced, as shown in Figure 1.

**Figure 2:**
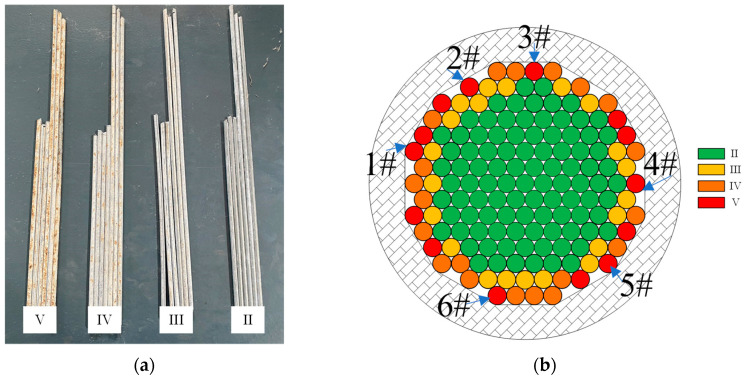
(**a**) Wire grade grading appearance. (**b**) Samples selected for the study.

The corrosion characteristics of the cable-stayed cable after service were checked based on the classification description of the corrosion degree given in the Technical Code for Bridge Maintenance of Highway Cable Structure System (JTGT 5122-2021) [44]. It was determined that the corrosion grade of the steel wire in the old cable was between II and V. The typical corrosion conditions corresponding to each corrosion grade are shown in Figure 2a. The proportion of steel wires with corrosion grade II is relatively large; the proportion of steel wires with corrosion grade V is relatively small. In this study, six steel wires with a corrosion grade of V at 1/2 of the cable were selected for a statistical study of corrosion. The position of the steel wires is shown in Figure 2b.

### 2.2. Methodology

**Figure 3:**
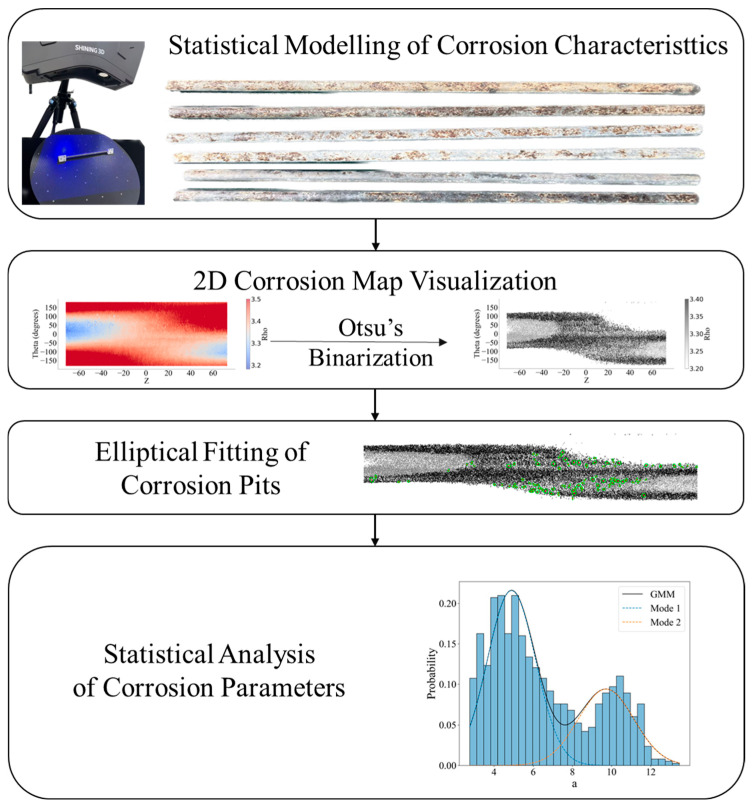
Flowchart of research idea.

Figure 3 shows the research idea of this paper. A brief description of the workflow is provided below.

After selecting the experimental specimen, the corroded steel wires underwent standardized de-rusting pre-treatment. Subsequent non-contact 3D laser scanning with metrologically certified accuracy was then performed to reconstruct morphological models, yielding quantitative surface characteristic datasets of the corrosion features and introducing the mass loss rate and volume loss rate to calculate the model error.

Based on the three-dimensional point cloud data, two-dimensional feature images of the corroded wire surfaces were constructed. The Otsu method was used to binarize the corrosion pit, then the corrosion pit was fitted with an ellipse on the basis of the processed image.

Connected pixel domains within the binarized images were identified, followed by minimum bounding ellipse fitting to delineate corrosion pits. Elliptical parameters were subsequently employed for dimensional characterization of the pits, enabling quantitative statistical analysis of the corrosion feature attributes.

Finally, the corrosion parameters were statistically analyzed.

### 2.3. Wire Rust Removal

**Figure 4:**
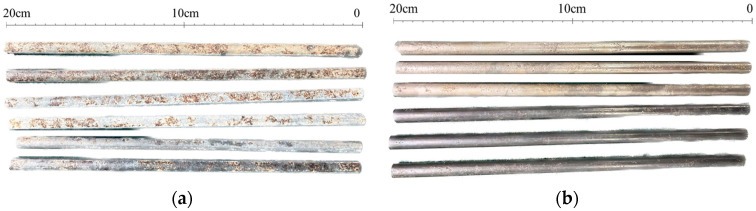
(**a**) Before rust removal. (**b**) After rust removal.

Corrosion of corroded wires is indicated for the removal of corrosion products according to international standards (ISO) [45] using a solution of 500 mL of hydrochloric acid (ρ = 1.19 g/mL) and 3.5 g of hexamethylenetetramine, diluted in distilled water to a total volume of 1000 mL. The corroded wire is soaked in solution for 10 min to remove the corrosion products from the high-strength wire. The wire is then rinsed with tap water, gently brushed with a clean steel wool ball, and then rinsed in deionized water. The wire sample is then naturally dried and cooled to room temperature. The effect before and after rust removal is given in Figure 4 (the sample number is 1#−6# from top to bottom). The mass of the sample is weighed on a highly accurate electronic balance before and after rust removal; the values are denoted as $m_{0}\;\mathrm{and}\;m$, respectively. The mass loss rate $loss_{m}$ is calculated according to Equation (1).

(1) $${loss}_{m}=\frac{m_{0}-m}{m_{0}}\times 100\%$$

Table 1 gives the mass before and after rust removal and the mass loss rate.

### 2.4. Three-Dimensional Scanning

**Figure 5:**
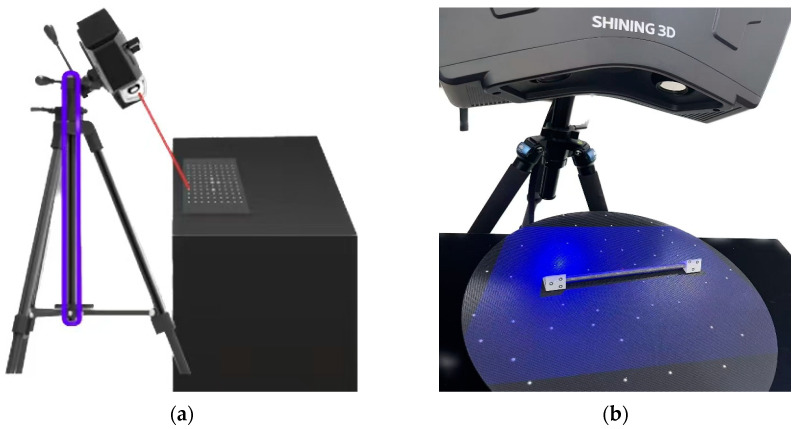
(**a**) 3D scanners. (**b**) Turntables and samples.

A 3D laser scanner (Figure 5a) with an accuracy range of 3–50 μm was used to obtain the corrosion parameters for a 150 mm steel wire cross section. Grip both ends of the wire securely with a custom swivel clamp to prevent any slippage that could affect alignment. Finally, the steel wire is placed on the turntable along the fixture, and the irregularly distributed marking points are fixed on the turntable and the fixture. Seamless alignment of the wire surface is achieved through the rotation of the fixture and turret (Figure 5b), resulting in point cloud data that accurately represents the surface characteristics of the corroded wire.

**Figure 6:**
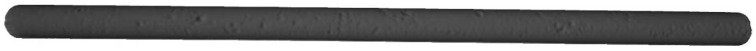
Three-dimensional modeling diagram.

The reconstructed surfaces from the 3D scans are stitched together by scanning after three fixture rotations to create a uniform and seamless representation of the entire corroded wire. Considering that the corroded steel wire can bend, the straightness of the corroded steel wire is restored by pre-stretching before the 3D scanning. Once the 3D laser scanner has obtained the point cloud data, the next step involves reconstructing the surface of the corroded wire. Eventually, the point cloud data undergoes pre-processing operations to optimize it for further processing. These operations include removing outliers, reducing noise, reconstructing coordinate systems, and applying filtering techniques. Subsequently, the pre-processed point cloud data was used to generate a mesh representation of the corroded wire surface in the form of a triangular grid by the software. The mesh includes interconnected triangular or polygonal elements of the geometry of the wire surface. Figure 6 shows an intuitive model of wire surface reconstruction. (In order to save space, only 5# sample is displayed.)

**Figure 7:**
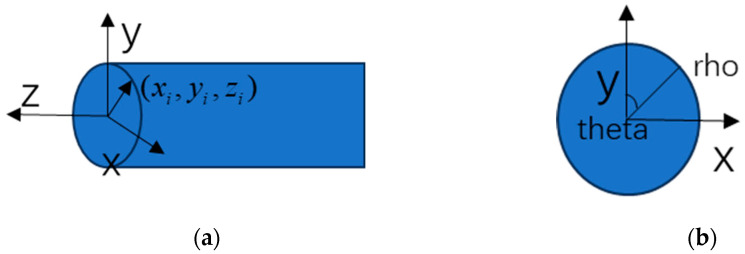
Schematic diagram of coordinates. (**a**) Longitudinal schematic of the coordinate system; (**b**) Transverse schematic of the coordinate system.

**Figure 8:**
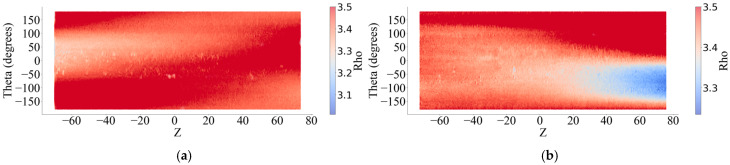

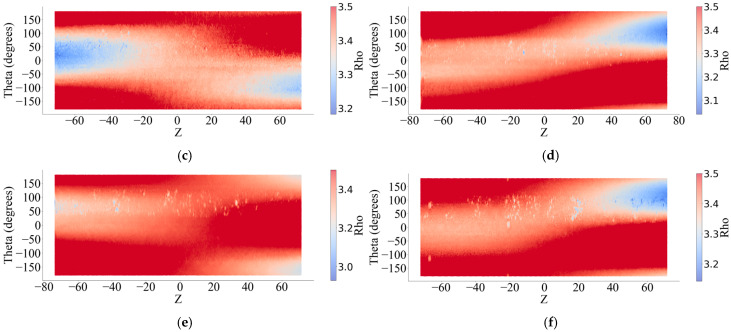
Two-dimensional corroded surfaces. (**a**) Sample No. 1. (**b**) Sample No. 2. (**c**) Sample No. 3. (**d**) Sample No. 4. (**e**) Sample No. 5. (**f**) Sample No. 6.

The point cloud data obtained by 3D laser scanning can be exported as $\left(x,\;y,\;z\right)$ data, where  x,y is the circumferential direction of the wire surface.  z represents the displacement of the middle of the wire along the length of the wire and the coordinate origin (Figure 7). According to Equation (2), the wire cross section is converted into a polar representation according to the data, and the axial coordinate z remains unchanged. Figure 8 shows the 2D corroded surface of the specimen after coordinate transformation.

(2) $$\left\{\begin{array}{c} z_{i}=z_{i}\\ {\;theta}_{i}=\arctan\left(\frac{x_{i}}{y_{i}}\right)\\ {\;rho}_{i}=\sqrt{x_{i}^{2}+y_{i}^{2}} \end{array}\right.$$

**Figure 9:**
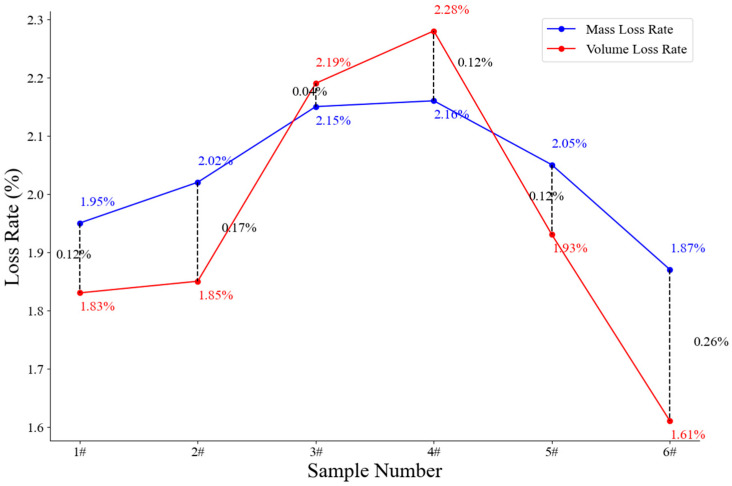
Radius loss rate vs. mass loss rate.

In order to verify the quality of the scan data, a method using the average radius loss rate equivalent to the mass loss rate was proposed to evaluate the scan model. Assuming that the density of corroded steel wire is uniformly distributed along the length direction, then its mass loss is proportional to the volume loss. In a two-dimensional plane such as in Figure 8, Equations (3) and (4) give a simplified formula for calculating the equivalent volume of steel wire, which ignores the transformation process because the changes, such as angle and radian transformation, are linear.

(3) $$V_{0}=|Z|\times |Theta|\times r$$

(4) $$loss_{v}=\frac{V_{0}-\sum |Z|\times |Theta|\times Rho}{V_{0}}\times 100\%$$

where $V_{0}$ is the theoretically equivalent original volume, Z is the axial coordinate, Theta is the circumferential angle, r  is the initial steel wire radius, and Rho  is the radius of the corroded steel wire at each point. Since  Z, Theta are equal at each point, the volume loss rate is proportional to the radius difference ratio and the volume loss rate is proportional to the mass loss rate. So, the average radius loss rate is used to evaluate the quality of the 3D scanning model by comparing it with the mass loss rate before and after rust removal. Equation (5) gives the calculation method of the average radius loss rate, and Figure 9 gives the comparison of the average radius loss rate with the mass loss rate. As can be seen from Figure 9, the steel wire model reconstruction through 3D laser scanning data is of good quality, and the error of each sample is not large.

(5) $$loss_{r}=\mathrm{mean}\;(\sum \frac{r-Rho}{r}\times 100\%)$$

## 3. Corrosion Feature Extraction

**Figure 10:**
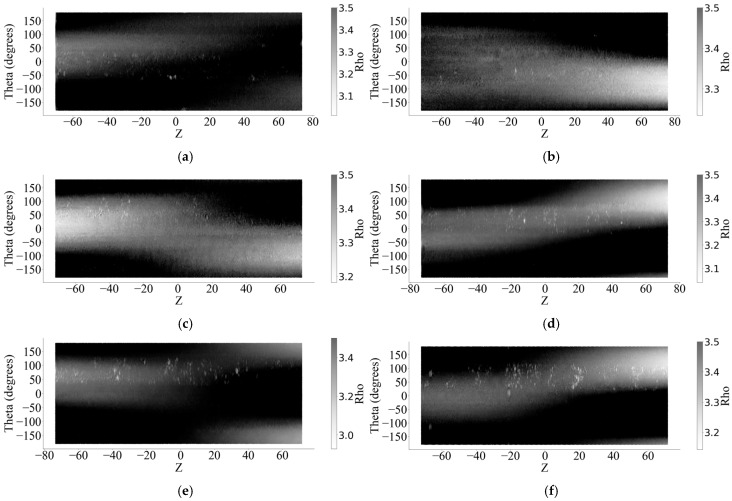
Grayscale image of a 2D corroded surface. (**a**) Sample No. 1. (**b**) Sample No. 2. (**c**) Sample No. 3. (**d**) Sample No. 4. (**e**) Sample No. 5. (**f**) Sample No. 6.

After the two-dimensional surface image of the corroded steel wire was obtained, the grayscale image of the corrosion pit size was obtained by the difference between the original radius and Rho. The grayscale image of the corrosion pit size was processed by the Otsu method. Figure 10 gives the grayscale image of the corrosion crater for each sample.

### 3.1. Binarization and the Otsu Method

In order to clearly distinguish and characterize the pits on the corroded surface from the background, a threshold t is given to separate the corrosion information from the background. If the gray value at the point $\;\left(z,theta\right)\;$ satisfies the discriminant condition $g\left(z,theta\right)\;<\;t$, it means that the point is a pit point and is colored black in the binary image. Otherwise, define the point as a white background.

In order to extract the pit area from the background surface, the Otsu method [46] was used to obtain the threshold. If the total number of pixels in a grayscale image is N, the grayscale range is $\left[0,\;L\;-\;1\right]$, the number of pixels in grayscale  i  is $n_{i}$, then the probability distribution of grayscale $\;p_{i}$ is

(6) $$p_{i}=\frac{n_{i}}{N},\;\overset{L-1}{\underset{i=0}{\sum }}p_{i}=1$$

The given threshold, t, divides the pixels into two classes, the foreground class $\;C_{0}$ and the background class $C_{1}$, with gray levels of $\left[0,\;t\right]$, $\left[t\;+\;1,\;L\;-\;1\right]$, respectively. The probability distributions and mean values for the two classes are

(7) $$\omega_{0}\left(t\right)=\overset{t}{\underset{i=0}{\sum }}p_{i},\;\omega_{1}\left(t\right)=\overset{L-1}{\underset{i=t+1}{\sum }}p_{i}=1-\omega_{0}\left(t\right)$$

(8) $$\mu_{0}\left(t\right)=\frac{\overset{t}{\underset{i=0}{\sum }}i\cdot p_{i}}{\omega_{0}\left(t\right)},\;\mu_{1}\left(t\right)=\frac{\overset{L-1}{\underset{i=t+1}{\sum }}i\cdot p_{i}}{\omega_{1}\left(t\right)}$$

Then, the overall mean and between-class variance of the whole image are defined as

(9) $$\mu_{T}=\overset{L-1}{\underset{i=0}{\sum }}i\cdot p_{i}=\omega_{0}\left(t\right)\mu_{0}\left(t\right)+\omega_{1}\left(t\right)\mu_{1}\left(t\right)$$

(10) $$\sigma_{B}^{2}\left(t\right)=\omega_{0}\left(t\right)[\mu_{0}\left(t\right)-\mu_{T}{]}^{2}+\omega_{1}\left(t\right)[\mu_{1}\left(t\right)-\mu_{T}{]}^{2}$$

Equation (9) is coupled with Equation (10) to obtain the simplified form of the variance between classes:

(11) $$\sigma_{B}^{2}\left(t\right)=\omega_{0}\left(t\right)\omega_{1}\left(t\right){\left[\mu_{0}\left(t\right)-\mu_{1}\left(t\right)\right]}^{2}$$

The optimal thresholds obtained by the Otsu method are

(12) $$t^{*}=\arg\max_{0\leq t\leq L-1}\sigma_{B}^{2}\left(t\right)$$

**Figure 11:**
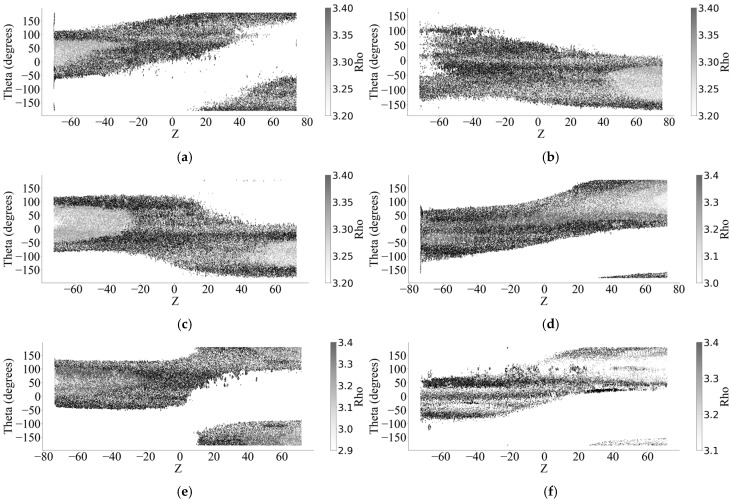
The Otsu method binarizes grayscale images. (**a**) Sample No. 1. (**b**) Sample No. 2. (**c**) Sample No. 3. (**d**) Sample No. 4. (**e**) Sample No. 5. (**f**) Sample No. 6.

Through binarization, a binary image of each sample can be obtained, and the original depth profile image can be divided into pit areas. Finally, the Otsu method is used to cluster the threshold of binary images and calculate the corrosion pits. Figure 11 shows all the binary images of the sample, where the black area indicates the location of the corrosion pit.

### 3.2. Elliptical Fitting of a Corrosion Pit

In this paper, an elliptical fitting corrosion pit based on the second-order central moment is used, which can effectively characterize the geometric characteristics of the corrosion pit. Firstly, the binary image $I_{b}$ is labeled with a connected domain, $\;L\;=\;label\left(I_{b}\right)$, where L   is the matrix of labels. The pixel area $A_{i}$ is calculated for each region $R_{i}$, and the effective domain $R_{i}^{'}$ is obtained by dividing the small region with an area less than the threshold $\tau_{A}$. For the points in the effective domain $R_{i}^{'}$, calculate their center coordinates, and calculate the Euclidean distance from each point to the center in the effective domain, where n  is the number of regional points and $\left(x_{k},\;y_{k}\right)$ are the pixel coordinates. The points whose distance is less than the threshold $\;\tau_{d}$ are filtered out to obtain the effective point set S.

(13) $$\mu_{x}=\frac{1}{n_{i}}\overset{n_{i}}{\underset{k=1}{\sum }}x_{k},\;\mu_{y}=\frac{1}{n_{i}}\overset{n_{i}}{\underset{k=1}{\sum }}y_{k}$$

(14) $$d_{k}=\sqrt{{\left(x_{k}-\mu_{x}\right)}^{2}+{\left(y_{k}-\mu_{y}\right)}^{2}}$$

The eigenvalues and eigenvectors of the covariance matrix of the effective point set S are used to characterize the axis length and direction angle of the fitted ellipse. The covariance matrix is expressed as

(15) $$\Sigma =\left[\begin{array}{cc} \sigma_{xx} & \sigma_{xy}\\ \sigma_{yx} & \sigma_{yy} \end{array}\right]$$

(16) $$\left\{\begin{array}{c} \sigma_{xx}=\frac{1}{n_{S}-1}\overset{n_{S}}{\underset{k=1}{\sum }}{\left(x_{k}-\mu_{x}\right)}^{2}\\ \sigma_{yy}=\frac{1}{n_{S}-1}\overset{n_{S}}{\underset{k=1}{\sum }}{\left(y_{k}-\mu_{y}\right)}^{2}\\ \sigma_{xy}=\sigma_{yx}=\frac{1}{n_{S}-1}\overset{n_{s}}{\underset{k=1}{\sum }}\left(x_{k}-\mu_{x}\right)\left(y_{k}-\mu_{y}\right) \end{array}\right.$$

The eigenvalues and eigenvectors of the covariance matrix ∑ are $\lambda_{1},\lambda_{2},\;\upsilon_{1},\;\mathrm{and}\;\;\upsilon_{2}$, respectively, where $\lambda_{1}\;>\;\lambda_{2}$. The length a and short axis b of the fitted ellipse and the direction angle are, respectively,

(17) $$a=2\sqrt{\lambda_{1}},\;b=2\sqrt{\lambda_{2}}$$

(18) $$\theta =\arctan2\left(\frac{v_{y1}}{v_{x1}}\right)\times \frac{180}{\pi }$$

**Figure 12:**
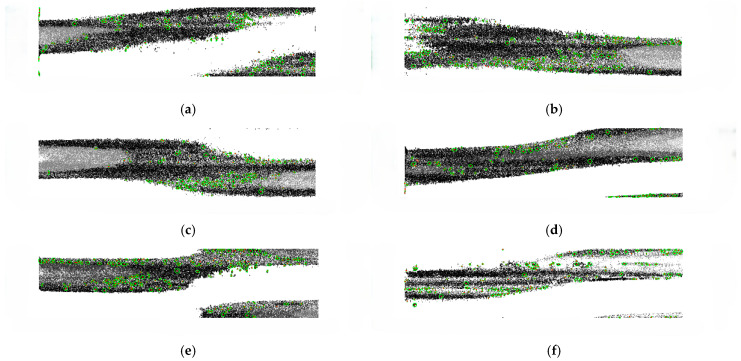
Elliptical fitting binary corrosion pit images. (**a**) Sample No. 1. (**b**) Sample No. 2. (**c**) Sample No. 3. (**d**) Sample No. 4. (**e**) Sample No. 5. (**f**) Sample No. 6.

The elliptical direction angle θ represents the angle between the major axis and the horizontal direction. When θ = 0, it means that the major axis of the elliptical is along the length of the corroded steel wire; when θ = 90, it means that the major axis of the elliptical is along the circumference of the steel wire. The ratio of the short axis to the long axis b/a defines the geometric characteristics of the wire corrosion pit, characterizing the sharpness of the corrosion pit. Lower values represent the sharper areas of the corrosion pit [31]. Figure 12 gives the corrosion pit results fitted according to the above method. The green regions indicate the elliptical corrosion pits obtained using the proposed method for corrosion pit fitting.

## 4. Wire Corrosion Characteristics

### 4.1. Basic Parameters

In order to characterize the corrosion pit, four key basic parameters were selected for statistical analysis: corrosion pit fitting the ellipse major axis length  a, corrosion pit fitting the ellipse minor axis length  b, corrosion pit direction angle  θ, and corrosion pit depth  d. Statistical analysis is carried out based on these basic parameters.

**Figure 13:**
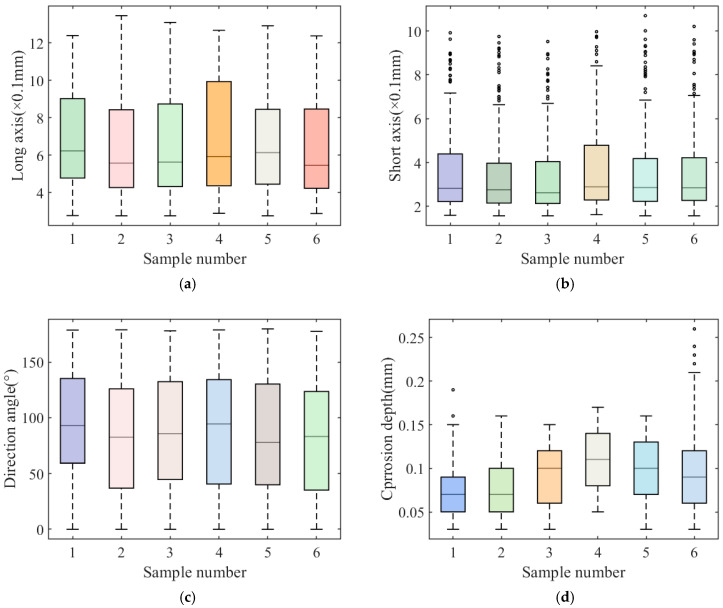
Basic parameters box diagram. (**a**) Elliptical corrosion pit long axis (×10^−1^ mm). (**b**) Elliptical corrosion pit short axis (×10^−1^ mm). (**c**) Direction angle of elliptical corrosion pit (°). (**d**) Elliptical corrosion pit depth (mm).

Figure 13a–d show the analysis results of the sample data, characterized by a 3D scan. These figures describe the distribution of the elliptical major axis, minor axis, directional angle, and depth of the corrosion pit. It can be seen from Figure 13a,b that the distribution ranges of the long and short axis of the corrosion pit in the six samples are roughly the same. The short axis of the corrosion pit has abnormal points greater than the upper limit, indicating that during the corrosion process. Meanwhile, the length distribution of the corrosion pit is relatively uniform, and the width development rate is irregular. Figure 13c shows that the corrosion pit direction angles of the six samples exist within the range of 0~180°; the upper and lower quartile positions of the six samples are basically the same. In Figure 13d, it can be seen that although the distribution of other geometric parameters of the corrosion pit is roughly the same, the distribution of the six samples with corrosion pit depth is relatively different. The median corrosion depth of the sample 4# is the largest. There are large abnormal depth data for sample 6# and sample 1#.

**Figure 14:**
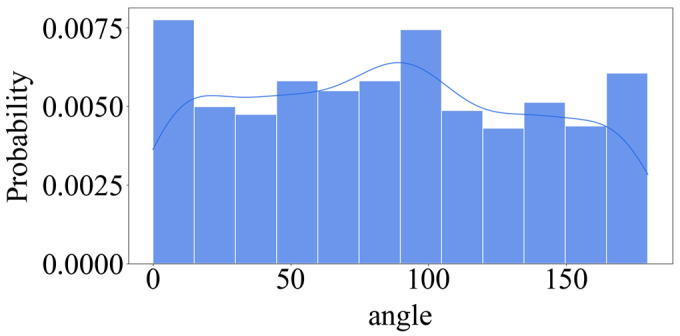
Theta distribution.

The six samples are the outermost steel wires of the cable, and the samples are not very different. In order to expand the statistical dataset to reduce statistical errors caused by accident or the small data volume, improving the accuracy of statistical results analysis, the 1#~6# sample data is combined to be called the enlarged dataset. The enlarged dataset is verified using the 1# sample data. Figure 14 shows the distribution of the directional angle of the corrosion pit. It can be seen that the directional angle is relatively uniform, and it is difficult to fit using the probability function. However, it is not difficult to find that the probability of the directional angle distributed near  0°  and 90°  is relatively high.

**Figure 15:**
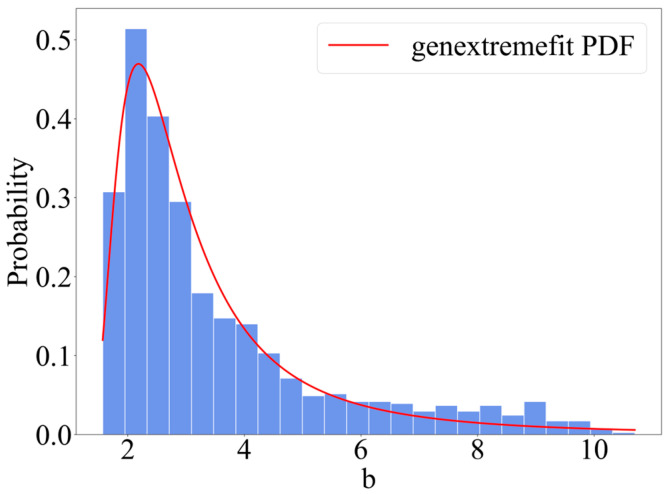
GEV fit of b.

The statistical distribution of parameter b can be fitted with a generalized extreme value (GEV) distribution. The K–S test does not reject the generalized extreme value distribution function at a 95% significance level. Figure 15 gives the fitting effect of parameter  b. As shown in the results, the width b of the corrosion pit is mainly concentrated at  0.2−0.3 mm; the larger the width, the smaller the probability. The generalized extreme value distribution can be written as Equation (19): k = −0.4859 is a shape parameter; μ = 2.4965  is a positional parameter; and σ = 0.8683 is a scale parameter.

(19) $$f(b|k,s,m)=\frac{1}{s}\exp\left[-{\left(1+\frac{k\left(b-m\right)}{s}\right)}^{-\frac{1}{k}}\right]{\left(1+\frac{k\left(b-m\right)}{s}\right)}^{-1-\frac{1}{k}}$$

The K–S test of parameters a and d rejects most commonly used single-function distribution fits at a 95% significance level. Therefore, the following Gaussian Mixture Model (GMM) is used to model and analyze the parameters a  and d.

(20) $$f\left(x\right)=\overset{K}{\underset{i=1}{\sum }}\omega_{i}\frac{1}{\sqrt{2\pi }\sigma_{i}}\exp\left[-\frac{{\left(x-\mu_{i}\right)}^{2}}{2\sigma_{i}^{2}}\right]$$

**Figure 16:**
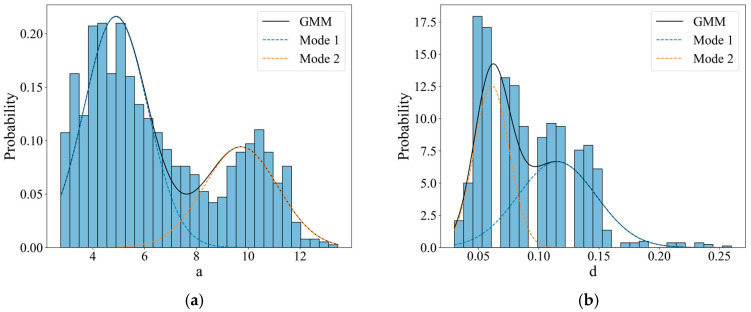
GMM distributions of (**a**) parameter a (×10^−1^ mm) and (**b**) parameter d (mm).

The distributions of geometric parameters a and d were observed in the mixed Gaussian distribution, as shown in Figure 16. The parameters of the related distribution function reported in Table 2. It is not difficult to see in Figure 16a that there are two peaks in the length distribution of corrosion pits (0.5 mm and 0.1 mm). From Figure 16b, it can be found that the depth of the corrosion pits is mainly concentrated below 0.15 mm.

### 4.2. Sharpness and Defect Parameters

The three-dimensional reconstruction model of the steel wire surface after corrosion can obtain the corrosion pit width and length ratio  b/a. This ratio reflects the sharpness of the corrosion pit and affects the stress concentration of the corroded steel wire. Therefore, studying the distribution of this ratio is conducive to analyzing the stress phenomenon of the corrosion wire. Murakami [47] proposed the defect parameter Φ  to measure the overall size of the corrosion pit. It can be solved by the following formula, where area is the outer envelope area of the irregular pit projected on the plane perpendicular to the stress axis, h is the depth of the corrosion pit, and c is half the width, i.e., c = d/2.

(21) $$\Phi =\sqrt{\mathrm{area}}=\sqrt{\frac{\pi }{2}hc}$$

**Figure 17:**
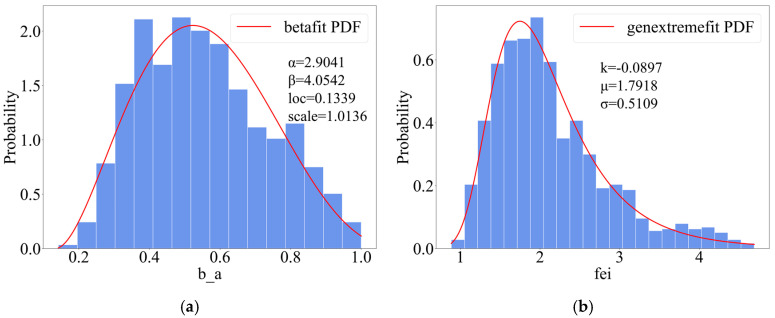
(**a**) Beta distribution of sharpness. (**b**) Defect parameter generalized extreme value distribution.

The K–S test found that at the 95% significance level, the aspect ratio does not reject the Beta distribution function, while the defect parameters do not reject the generalized extreme value distribution. Therefore, the aspect ratio conforms to the Beta distribution and the defect parameters conform to the generalized extreme value distribution. The Beta distribution function is as Equation (22), where α and β represent shape parameters,  loc represents positional parameters, and scale represents scale parameters. The distribution diagram of the aspect ratio and defect parameters is shown in Figure 17.

(22) $$f\left(x;a,b,loc,scale\right)=\frac{\Gamma \left(a+b\right)}{\Gamma \left(a\right)\Gamma \left(b\right)}\cdot \frac{{\left(x-loc\right)}^{a-1}\cdot {\left(loc+scale-x\right)}^{b-1}}{scale^{a+b-1}}$$

## 5. Discussion

In previous studies, researchers have predominantly used accelerated corrosion to simulate the corrosion conditions of cable-stayed cables and conducted probabilistic modeling of the corrosion pit conditions using three-dimensional scanning equipment [21,31,34,48,49,50,51,52]. However, the corrosion conditions during service are different from the accelerated corrosion test conditions. In order to more accurately describe the corrosion conditions of steel wires in service, this paper conducts statistical modeling of corrosion pits after 3D scanning corrosion wires that have been in service for 27 years. The results are indeed different from the probability distributions from accelerated corrosion.

**Figure 18:**
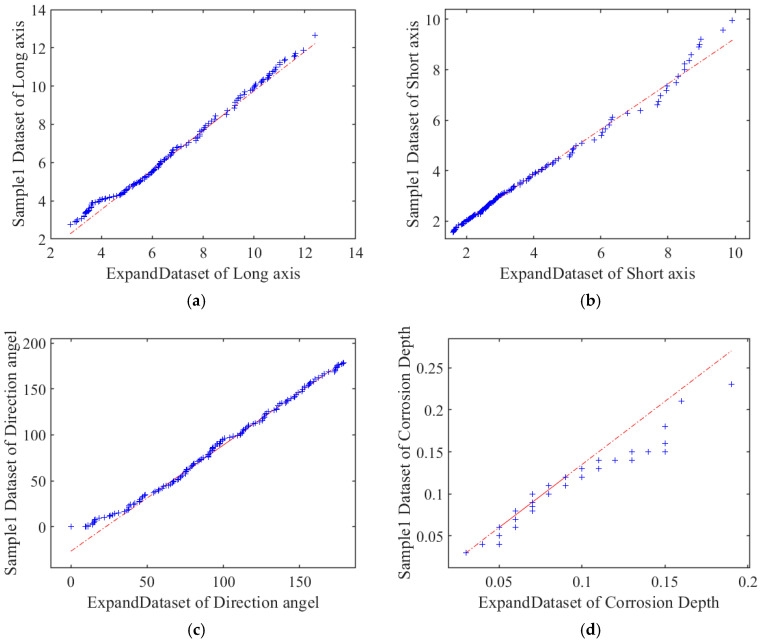
The expanded dataset and the 1# dataset Quantile–Quantile diagram of four basic parameters. (**a**) Elliptical corrosion pit long axis ($\times 10^{-1}\;\mathrm{mm}$). (**b**) Elliptical corrosion pit short axis ($\times 10^{-1}\;\mathrm{mm}$). (**c**) Direction angle of elliptical corrosion pit (°). (**d**) Elliptical corrosion pit depth (mm).

The datasets were merged in Section 4.1. Figure 18 displays the Quantile–Quantile plot comparing the expanded dataset and Sample 1. As shown in Figure 18a, the data points for smaller long-axis dimensions exhibit high density and closely align with the diagonal, indicating strong agreement between the datasets within this range. Conversely, for larger dimensions, points show increasing dispersion and deviation from the diagonal. For smaller minor-axis dimensions in Figure 18b, the data points exhibit high density and near-perfect diagonal alignment, indicating exceptional morphological similarity between the extended and sample datasets. As the dimensions increase, points show marginally elevated dispersion yet maintain minimal deviation from the diagonal, remaining within statistical confidence bounds. This indicates significant stochasticity in large-feature generation.

Such deviations likely arise from the greater complexity of actual corrosion processes, where large pit formation involves anisotropic growth influenced by multiple factors. Consequently, elliptical fitting demonstrates limitations in precisely capturing these pits’ geometric features. Collectively, this demonstrates that despite stochastic variations during data expansion, the expanded dataset maintains the underlying distributional properties of the sample data.

As shown in Figure 18c, data points cluster uniformly near the diagonal across the primary angular range (0–150°), indicating a stable linear correlation. Specifically, within this interval, as the extended dataset’s direction angle increases along the horizontal axis, the sample dataset’s angle values on the vertical axis show a stable linear-following trend, which visually and quantitatively reflects a stable linear correlation between the two datasets. This stable distribution indicates that, across most orientations, the angular probability distributions of the extended dataset and the sample dataset are consistent. However, within 150–180°, points display increased sparsity with intermittent diagonal deviations. This likely stems from the intrinsically low formation probability of corrosion pits at these orientations during actual corrosion, resulting in statistically insufficient representation in the sample data. Consequently, data expansion faces limitations in precisely replicating the distributional characteristics of these underrepresented morphological features.

Figure 18d illustrates the comparison of corrosion pit depths between the sample dataset and the expanded dataset. The blue scatter points represent the paired depth values from the two datasets, and the red dashed diagonal line denotes the ideal state of complete morphological congruence (i.e., identical depth values in both datasets). At shallow depths (e.g., below 0.1 units on the horizontal axis), the blue points cluster extremely closely to the red diagonal. This near-perfect alignment visually demonstrates that, for shallow corrosion pits, the morphological characteristics captured by the two datasets are highly consistent. In other words, the way shallow pits form and are measured shows exceptional congruence, with the depth values from the sample dataset and the expanded dataset matching closely. However, when the depth exceeds 0.1 units, a clear divergence emerges.

Integrated analysis across all four figures reveals strong statistical concordance in distributional characteristics between the extended and sample datasets. Consequently, leveraging the extended dataset for statistical analyses is methodologically justified to enhance population robustness and mitigate the sampling errors inherent to limited original datasets.

It should be noted that the modeling method of corrosion pits can be used for any degree of corrosion in high-strength steel wires. However, many tiny pits will connect to each other during the corrosion process, forming polygons of varying sizes. Therefore, there may be some errors in ellipse approximation. This study only studied six steel wires with rust grade V  at the 1/2 position of the cable, and all of them were the outermost layers of the cable. Research on steel wires with different levels of corrosion, different cable positions, and in different layers of the cable needs to be expanded.

## 6. Conclusions

This study investigated the corrosion characteristics of high-strength steel wire after 27 years of service, focusing on features such as corrosion pit length, width, depth, aspect ratio, and defect parameters. These features were extracted using three-dimensional point cloud data and statistically analysed to develop probabilistic models for corrosion characterization. The key conclusions and future directions are outlined below:

**Accurate Feature Extraction Using 3D Scanning:** High-resolution 3D laser scanning was used to capture the surface point cloud data of corroded steel wire. The Otsu binarization method was applied to the reconstructed surface, enabling effective identification and quantification of corrosion pits. The proposed method achieved an estimated error of only 0.2%, demonstrating its precision for extracting corrosion features.

**Statistical Modeling of Corrosion Pit Parameters:** Statistical analysis was performed on corrosion pit parameters: length (a), width (b), depth (d), direction angle (θ), aspect ratio (b/a), and defect parameter (Φ). The K–S test at a 95% confidence level indicated that

- The direction angle (θ) does not conform to any standard probability distribution.
- The width (b) and defect parameter (Φ) follow a generalized extreme value distribution.
- The aspect ratio (b/a) fits a Beta distribution.
- The pit length (a) and depth (d) are well described by a Gaussian mixture model.
- These findings provide a valuable statistical foundation for characterizing the distribution of corrosion pits on in-service wires.

**Comparison with Accelerated Corrosion Data:** A comparison with previous studies based on accelerated corrosion tests revealed that in-service corrosion environments are more complex, leading to greater variability in pit parameters. This underscores the need for in situ data to capture realistic corrosion behavior.

**Development of a Corrosion Pit Probability Model:** A probabilistic model was established based on real corrosion data from service-exposed wires. This model offers a meaningful reference for evaluating the condition of cable-stayed bridge cables and contributes to reliability-based maintenance and safety assessment.

Despite its contributions, this study has several limitations. The analysis was based on a limited number of samples from a single service environment, which may restrict the generalizability of the findings. Only surface corrosion features were considered, and the mechanical implications of corrosion—such as its impact on static strength and fatigue performance—were not explored. Future research should expand the dataset to include steel wires exposed to diverse climates, corrosion conditions, and structural layers. In addition, further studies should examine the mechanical effects of different corrosion patterns and consider integrating advanced sensing or machine learning techniques to enhance corrosion detection and predictive modeling.

## Figures and Tables

**Table 1:** Loss of quality before and after rust removal.

Specimen Number	Mass Before Rust Removal $m_{0}\left(g\right)$	Mass After Rust Removal $m\left(g\right)$	Quality Loss Rate $loss_{m}$ (%)
1#	56.157	55.062	1.95
2#	58.392	57.215	2.02
3#	57.144	55.913	2.15
4#	57.311	56.073	2.16
5#	57.257	56.083	2.05
6#	56.394	55.240	2.05

**Table 2:** Distribution function parameters.

	k	$\omega_{i}$	$\mu_{i}$	$\sigma_{i}$
a	2	0.663880	4.892641	1.226885
		0.336120	9.721610	1.425726
d	2	0.540497	0.115243	0.032355
		0.459503	0.060918	0.014593

## Data Availability

The original contributions presented in the study are included in the article. Further inquiries can be directed to the corresponding authors.
